# Supplementary material for: Similarities and Differences of Hsp70, hsc70, Grp78 and Mortalin as Cancer Biomarkers and Drug Targets
Source: Cells. 2021 Nov 3;10(11):2996. doi: 10.3390/cells10112996 (PMC8616428; doi:10.3390/cells10112996)
Supplement: Supplementary file 1 [file cells-10-02996-s001.zip › Supplementary Table S1.pdf]

Gene	Full Name	Aliases	Location
**HSPA1A**	Heat Shock Protein Family A (Hsp70) Member 1A	HSPA1, HSP72, **HSP70-1**, DnaK-Type Molecular Chaperone HSP70-1, Epididymis Secretory Sperm Binding Protein, DnaK-Type Molecular Chaperone HSP70-1, Heat Shock 70 KDa Protein 1A/1B, Heat Shock 70 KDa Protein ½, Heat Shock 70 KDa Protein B, Heat Shock 70 KDa Protein 2, HSP70-1/HSP70-2 HSP70I, HSP70-2, HSP70.1, HSP70.2, HSP70-1A, HEL-S-103, HSX70, HEL-S-103, Hsp, HSP68, Hsp7, HSP10.3, HSP70-1A, HSP70-2, hsp70A1, Hspa2, HSX70, IHSP70	Aggresome, blood microparticle, centriole, centrosome, cytoplasm, cytosol, endoplasmic reticulum, extracellular exosome, extracellular region, ficolin-1-rich granule lumen, adhesion, inclusion body, mitochondria, nuclear speck, nucleoplasm, nucleus
**HSPA1B**	Heat Shock Protein Family A (Hsp70) Member 1B	HSP72, **HSP70-2**, HSX70, HSP70-1, HSP70.1, HSP70.2, HSP70-1B, HSX70, Heat Shock 70 KDa Protein 1A/1B, Heat Shock 70 KDa Protein ½, Heat Shock 70 KDa Protein 1A, Heat Shock 70 KDa Protein 1, HSP70-1/HSP70-2, HSP70.1/HSP70.2, HSP70-3, HEL-S-103, HSX70, HEL-S-103, Hsp, HSP68, Hsp7, HSP10.3, HSP70-1B, HSP70-2, hsp70B1, Hspa2, HSX70, IHSP70	Aggresome, blood microparticle, centriole, centrosome, cytoplasm, cytosol, endoplasmic reticulum, extracellular exosome, extracellular region, ficolin-1-rich granule lumen, adhesion, inclusion body, mitochondria, nuclear speck, nucleoplasm, nucleus
**HSPA1L**	Heat Shock Protein Family A (Hsp70) Member 1 Like	HSP70-HOM, **HSP70-1L**, **Hsp70-1t**, HSPA1L, HSP70T, Epididymis Secretory Sperm Binding Protein, hum70t, Heat Shock 70 KDa Protein 1-Like, Heat Shock 70 KDa Protein 1-Hom, Heat Shock 70 KDa Protein 1L, Hum70t, Msh, Msh5, HSP70-HOM	Blood microparticle, cell body, cytoplasm, cytosol, nucleoplasm, nucleus, plasma membrane, zona pellucida receptor complex
**HSPA2**	Heat Shock Protein Family A (Hsp70) Member 2	HSP70-2, HSP70-3, Heat Shock-Related 70 KDa Protein 2, Epididymis Secretory Sperm Binding Protein, Heat Shock-Related 70 KDa Protein 2, Heat Shock 70kDa Protein 2, HSPA70.2, HSPT70, Hst70	CatSper complex, blood microparticle, cell surface, cytoplasm, cytosol, extracellular exosome, male ger cell nucleus, meiotic spindle, membrane, nucleus, plasma membrane, synaptonemal complex
**HSPA3**	No Information		
**HSPA4**	Heat Shock Protein Family A (Hsp70) Member 4	HS24/P52, HSPH2, Hsp70 RY, Epididymis Secretory Sperm Binding Protein Li 5a, APG-2, HS24/P52, RY, HEL-S-5a, HS24/P52, Heat Shock 70-Related Protein APG-2, Heat Shock 70 KDa Protein 4, Al319151, APG, Hsp1, Hsp110, Hsp7, Hsp70, Hsp70RY, irp94, mKlAA4025	Cytosol, extracellular exosome, mitochondria, nucleus
**HSP4L**	Heat Shock Protein family A (Hsp70) member 4 like	AC146980.1, Al461691, APG, APG-1, heat shock protein 4 like, heat shock protein family A (hsp70) member 4 like, HSPH3, Os, OSP94	Cytoplasm, cytosol, exosomes, Nucleus
**HSPA5**	Heat Shock Protein Family A (Hsp70) Member 5	Binding immunoglobulin protein **(BiP),** 78kDa glucose-regulated protein (**GRP78**), Mif-2, Heat Shock 70kDa Protein 5 (Glucose-Regulated Protein, 78kDa), HEL-S-89n, Endoplasmic Reticulum Chaperone BiP, Glucose-Regulated Protein, 78kDa, HSP70 Family Protein 5, Heat Shock 70kD Protein 5 (Glucose-Regulated Protein, 78kD), Endoplasmic Reticulum Lumenal Ca(2+)-Binding Protein Grp78, Epididymis Secretory Sperm Binding Protein Li 89n, EC 3.6.4.10, HEL-S-89n, GRP-78, AL022860, AU019543, B, baffl, baffled, D2Wsu141, D2Wsu141e, D2Wsu17, D2Wsu17e, Grp, heat shock protein 5, Hsce7, Hsce70, HSP70-5, IDBP2, immunoglobulin heavy chain binding, mB, mBiP, Sez, SEZ-7	Cell surface, cytoplasm, cytosol, endoplasmic reticulum, endoplasmic reticulum chaperone process, endoplasmic reticulum lumen, endoplasmic reticulum- Golgi intermediate compartment, extracellular exosome, focal adhesion, endoplasmic reticulum membrane, intracellular membrane- bounded organelle, melanosome

**HSPA5P1**	Heat Shock Protein Family A (Hsp70) Member 5 pseudogene 1	RP11 114L91	unknown
**HSPA6**	Heat Shock Protein Family A (Hsp70) Member 6	**HSP70B'**, Heat Shock 70kDa Protein 6 (HSP70B'), Heat Shock 70 KDa Protein 6, Epididymis Secretory Sperm Binding Protein, Heat Shock 70 KDa Protein B', IDBP4	Blood microparticle, centriole, cytoplasm, cytosol, extracellular exosome, extracellular region, ficolin-1-rich granule lumen, nucleus, plasma membrane, secretory granule lumen
**HSPA7**	Heat Shock Protein Family A (Hsp70) Member 7 (Pseudogene)	HSP70B, pseudogene of HSPA6 [9], Heat Shock 70 KDa Protein B, Heat Shock 70kDa Protein 7 (HSP70B), Heat Shock Protein Family A (Hsp70) Member 7, Heat Shock 70 KDa Protein B	Blood microparticle, cytoplasm, cytosol, extracellular exosome, nucleus, plasma membrane
**HSPA8**	Heat Shock Protein Family A (Hsp70) Member 8	**HSC70**, Constitutive Heat Shock Protein 70, HSP73, LAP1, HSC54, HSC71, HSP71, HSP73, LAP-1, NIP71, HEL-33, HSPA10, HEL-S-72p, Epididymis Luminal Protein 33, Heat Shock Cognate Protein 54, Heat Shock Cognate 71 KDa Protein, Heat Shock 70kDa Protein 8, Heat Shock 70kd Protein 10, Heat Shock 70kD Protein 8, LPS-Associated Protein 1, N-Myristoyltransferase Inhibitor Protein 71, Lipopolysaccharide-Associated Protein 1, Epididymis Secretory Sperm Binding Protein Li 72p, EC 3.6.4.10, 2410008N15Rik, DnaK-type chaperone hsp72/uncoating ATPase, HEL-33, HS7C, HSC54, hsc7, HSC70, Hsc73, Hsp, HSP70 7c, HSP7c, Hsp 8, HSPA10, IDBP1, LOC624853, Stro-1	Prp19 complex, autophagosome, blood microparticle, chaperone complex, clathrin-sculpted gamma- aminobutyric acid transport vesicle membrane, cytoplasm, cytosol, dendrite, extracellular exosome, extracellular region, ficolin-1-rich granule lumen, focal adhesion, glutamatergic synapse, glycinergic synapse, late endosome, luminal side of lysosomal membrane
**HSPA9**	Heat Shock Protein Family A (Hsp70) Member 9	**GRP75, Mortalin**, Mortalin2, MOT, MOT2, Mot-2, P66-Mortalin, Mortalin, Perinuclear, HEL-S-124m, MTHSP70, MTHSP75, Mthsp75, SIDBA4, CRP40, EVPLS Peptide-Binding Protein 74 (PBP74), CSA, SAAN, CRP40, EVPLS, HSPA9B, SIDBA4, MTHSP75, Epididymis Secretory Sperm Binding Protein Li 124m, Hs, Hsc7, Hsc74, Hsp, HSP74, Hsp74a, Hspa9a, IDBP-3, Mitochondrial stress-70, mo, mthsp, PBP, PBP74, SAAN, SIDBA4, Stress-70	MIB complex, SAM complex, cytoplasm, extracellular exosome, focal adhesion, mitochondrial matrix, mitochondrial nucleoid, mitochondrion, nucleolus
**HSPA9P1**	Heat Shock Protein family A (Hsp70) member 9 pseudogene 1	heat shock protein family A (Hsp70) member 9 pseudogene 1, HSPA9BP, HSPA9P	unknown
**Hspa9-ps1**	heat shock protein 9, pseudogene 1	AL669898.5, heat shock protein 9, pseudogene 1, Hsc74-, Hsc74-ps1, Hsp, Hsp7, Hsp74, Hsp74b, Hsp74-ps1, Hspa9b, Mitochondrial stress-70	cytoplasm
**HSPA10**	See HSPA8		
**HSPA11**	No Information		
**HSPA12A**	Heat Shock Protein Family A (Hsp70) Member 12A	KIAA0417, mKIAA0417, FLJ13874, Heat Shock 70 KDa Protein 12A, 1700063D12Rik, Al118035, Al840429, AW553406, D5Mgi4, D5Mgi40, Gm19925	Cytoplasm, extracellular exosome, nucleus

**HSPA12B**	Heat Shock Protein Family A (Hsp70) Member 12B	C20orf60, DJ1009E24.2, Chromosome 20 Open Reading Frame 60, Heat Shock 70kD Protein 12B, 2700081N06Rik	Unknown
**Hspa12b**	Heat Shock Protein Family A (Hsp70) member 12B	heat shock protein family A (Hsp70) member 12B	Unknown
**HSPA13**	Heat Shock Protein Family A (Hsp70) Member 13	STCH, Testis Secretory Sperm-Binding Protein Li 199a, Microsomal Stress 70 Protein ATPase Core, Stress 70 Protein Chaperone, Microsome-Associated, 60kD, Stress-70 Protein Chaperone Microsome-Associated 60 KDa Protein, 1600002l10Rik, AV006182, B230217N24Rik, heat shock protein 70 family, Stc, STRESS 70 protein CHAPERONE	Cytoplasm, endoplasmic reticulum, extracellular exosome, intracellular membrane-bounded organelle, mitochondria, microsome
**HSPA14**	Heat Shock Protein Family A (Hsp70) Member 14	HSP70L1, HSP60, HSP70-4, Heat Shock Protein Hsp70-Related Protein, HSP70-Like Protein 1, Heat Shock Protein HSP60, Heat Shock 70 KDa Protein 14, heat shock protein 14, hsr.1, LOC100503996, LOC100861945, NST-1	Cytoplasm, cytosol, plasma membrane, ribosome, membrane

Citations

[1] M. Daugaard, M. Jaattela, M. Rohde, Hsp70-2 is required for tumor cell growth and survival, Cell Cycle, 4 (2005) 877-880.

[2] M. Daugaard, M. Rohde, M. Jaattela, The heat shock protein 70 family: Highly homologous proteins with overlapping and distinct functions, FEBS Lett, 581 (2007) 3702-3710.

[3] J.H. Lee, Y.S. Han, Y.M. Yoon, C.W. Yun, S.P. Yun, S.M. Kim, H.Y. Kwon, D. Jeong, M.J. Baek, H.J. Lee, S.J. Lee, H.J. Han, S.H. Lee, Role of HSPA1L as a cellular prion protein stabilizer in tumor progression via HIF-1alpha/GP78 axis, Oncogene, 36 (2017) 6555-6567.

[4] D.R. Sojka, A. Gogler-Piglowska, N. Vydra, A.J. Cortez, P.T. Filipczak, Z. Krawczyk, D. Scieglinska, Functional redundancy of HSPA1, HSPA2 and other HSPA proteins in non-small cell lung carcinoma (NSCLC); an implication for NSCLC treatment, Sci Rep, 9 (2019) 14394.

[5] J. Wang, J. Lee, D. Liem, P. Ping, HSPA5 Gene encoding Hsp70 chaperone BiP in the endoplasmic reticulum, Gene, 618 (2017) 14-23.

[6] J.P. Lievremont, R. Rizzuto, L. Hendershot, J. Meldolesi, BiP, a major chaperone protein of the endoplasmic reticulum lumen, plays a direct and important role in the storage of the rapidly exchanging pool of Ca2+, J Biol Chem, 272 (1997) 30873-30879.

[7] C.A.S. Deane, I.R. Brown, Differential Targeting of Hsp70 Heat Shock Proteins HSPA6 and HSPA1A with Components of a Protein Disaggregation/Refolding Machine in Differentiated Human Neuronal Cells following Thermal Stress, Front Neurosci, 11 (2017) 227.

[8] E.J. Noonan, R.F. Place, C. Giardina, L.E. Hightower, Hsp70B' regulation and function, Cell Stress Chaperones, 12 (2007) 219-229.

[9] A.J. Parsian, J.E. Sheren, T.Y. Tao, P.C. Goswami, R. Malyapa, R. Van Rheeden, M.S. Watson, C.R. Hunt, The human Hsp70B gene at the HSPA7 locus of chromosome 1 is transcribed but non-functional, Biochim Biophys Acta, 1494 (2000) 201-205.

[10] T. Liu, C.K. Daniels, S. Cao, Comprehensive review on the HSC70 functions, interactions with related molecules and involvement in clinical diseases and therapeutic potential, Pharmacol Ther, 136 (2012) 354-374.

[11] N. Shan, W. Zhou, S. Zhang, Y. Zhang, Identification of HSPA8 as a candidate biomarker for endometrial carcinoma by using iTRAQ-based proteomic analysis, Onco Targets Ther, 9 (2016) 2169-2179.

[12] T. Liu, K. Krysiak, C.L. Shirai, S. Kim, J. Shao, M. Ndonwi, M.J. Walter, Knockdown of HSPA9 induces TP53-dependent apoptosis in human hematopoietic progenitor cells, PLoS One, 12 (2017) e0170470.

[13] C. Peng, P. Yang, Y. Cui, M. He, L. Liang, Y. Di, HSPA9 overexpression inhibits apoptin-induced apoptosis in the HepG2 cell line, Oncol Rep, 29 (2013) 2431-2437.
