# Supplementary material for: Similarities and Differences of Hsp70, hsc70, Grp78 and Mortalin as Cancer Biomarkers and Drug Targets
Source: Cells. 2021 Nov 3;10(11):2996. doi: 10.3390/cells10112996 (PMC8616428; doi:10.3390/cells10112996)
Supplement: Supplementary file 1 [file cells-10-02996-s001.zip › Supplementary Table S3.pdf]

Supplemental Table S3. Top 5 Diseases and Biological Functions Associated with Hsp70-1, hsc70, Grp78 and mortalin.

Name	p-value range	# Molecules
**Hsp70-1**		
Cancer	1.37E-21 - 5.25E-54	560
Infectious Diseases	2.42E-24 - 8.83E-52	210
Organismal Injury and Abnormalities	1.60E-21 - 8.70E-49	563
Reproductive System Disease	2.38E-22 - 4.83E-44	431
Endocrine System Disorders	2.24E-37 - 3.92E-41	486
**Hsc70**		
Cancer	4.73E-22 - 1.13E-93	1303
Organismal Injury and Abnormalities	4.75E-22 - 1.13E-93	1315
Endocrine System Disorders	3.30E-22 - 3.48E-79	1144
Gastrointestinal Disease	3.30E-22 - 5.77E-74	1201
Infectious Diseases	2.83E-25 - 2.98E-66	396
**Grp78**		
Cancer	2.21E-19 - 3.11E-70	934
Organismal Injury and Abnormalities	2.21E-19 - 3.11E-70	939
Gastrointestinal Disease	2.41E-19 - 1.47E-56	872
Endocrine System Disorders	5.54E-20 - 6.06E-54	818
Reproductive System Disease	2.21E-19 - 3.17E-44	711
**Mortalin**		
Cancer	4.82E-16 - 1.27E-37	473
Organismal Injury and Abnormalities	4.82E-16 - 2.20E-37	476
Reproductive System Disease	1.96E-16 - 9.22E-37	372
Endocrine System Disorders	4.47E-31 - 1.97E-33	416
Renal and Urological Disease	1.74E-16 - 2.05E-33	195
