# Supplementary material for: Similarities and Differences of Hsp70, hsc70, Grp78 and Mortalin as Cancer Biomarkers and Drug Targets
Source: Cells. 2021 Nov 3;10(11):2996. doi: 10.3390/cells10112996 (PMC8616428; doi:10.3390/cells10112996)
Supplement: Supplementary file 1 [file cells-10-02996-s001.zip › Supplementary Table S4.pdf]

Supplementary Table S4. Top 5 Physiological System Development and Functions Associated with Hsp70-1, hsc70, Grp78 and mortalin.

Name	p-value range	# Molecules
**Hsp70-1**		
Organismal Survival	1.87E-32 - 2.25E-67	283
Embryonic Development	1.76E-21 - 2.57E-45	278
Organismal Development	1.76E-21 - 2.57E-45	332
Connective Tissue Development and Function	4.07E-22 - 1.71E-42	173
Tissue Morphology	1.60E-21 - 2.59E-42	267
**Hsc70**		
Organismal Survival	1.45E-32 - 2.86E-77	530
Embryonic Development	3.28E-23 - 6.05E-49	487
Tissue Morphology	4.21E-22 - 2.89E-42	432
Organismal Development	1.39E-22 - 1.24E-39	647
Connective Tissue Development and Function	1.99E-23 - 3.22E-39	215
**Grp78**		
Organismal Survival	1.39E-29 - 1.30E-62	395
Tissue Morphology	1.73E-19 - 9.71E-51	396
Embryonic Development	1.55E-19 - 1.12E-44	372
Connective Tissue Development and Function	1.87E-19 - 1.09E-42	268
Tissue Development	2.20E-19 - 1.09E-42	415
**Mortalin**		
Embryonic Development	2.86E-16 - 2.20E-49	209
Organismal Survival	9.73E-28 - 6.70E-46	224
Tissue Morphology	2.32E-16 - 4.71E-38	217
Lymphoid Tissue Structure and Development	6.28E-17 - 4.97E-34	155
Hematological System Development and Function	4.60E-16 - 9.32E-33	183
