# Supplementary material for: Similarities and Differences of Hsp70, hsc70, Grp78 and Mortalin as Cancer Biomarkers and Drug Targets
Source: Cells. 2021 Nov 3;10(11):2996. doi: 10.3390/cells10112996 (PMC8616428; doi:10.3390/cells10112996)
Supplement: Supplementary file 1 [file cells-10-02996-s001.zip › Supplementary Table S5.pdf]

Supplementary Table S5. Top 5 Molecular and Cellular Functions Associated with Hsp70-1, hsc70, Grp78 and mortalin.

Name	p-value range	# Molecules
**Hsp70-1**		
Cell Death and Survival	1.98E-21 - 4.81E-91	368
Cell Cycle	1.91E-21 - 3.34E-73	281
Gene Expression	3.01E-32 - 1.25E-71	278
Cellular Development	2.25E-21 - 2.65E-65	342
Cellular Growth and Proliferation	2.25E-21 - 2.65E-65	328
**Hsc70**		
Cell Death and Survival	7.37E-23 - 8.29E-117	737
Cell Cycle	1.22E-23 - 1.08E-85 4	56
Gene Expression	5.09E-24 - 6.20E-85	523
Cellular Development	2.99E-22 - 1.79E-75	597
Cellular Growth and Proliferation	2.99E-22 - 1.79E-75	611
**Grp78**		
Cell Death and Survival	1.98E-19 - 9.32E-94	529
Cell Cycle	1.21E-19 - 1.99E-63	328
Protein Synthesis	1.51E-19 - 1.46E-60	237
Cellular Development	2.20E-19 - 1.02E-57	464
Cellular Growth and Proliferation	1.76E-19 - 1.02E-57	452
**Mortalin**		
Cell Death and Survival	3.77E-16 - 3.43E-78	320
Cell Cycle	5.20E-16 - 1.64E-51	222
Cellular Development	2.86E-16 - 4.13E-51	275
Cellular Growth and Proliferation	1.73E-16 - 4.13E-51	274
Gene Expression	9.98E-29 - 8.91E-47	216
